# Utilizing two different sustainable and green spectrophotometric approaches using derivative ratio spectra for the determination of a ternary severely overlapped mixture: application to veterinary formulation

**DOI:** 10.1186/s13065-024-01220-4

**Published:** 2024-06-26

**Authors:** Hind A. Abdullatif, Mohammed Abdelkawy, Maha Kamal, Nesma M. Fahmy

**Affiliations:** 1 Pharmaceutical Chemistry Department, Faculty of Pharmacy, Ahram Canadian University, Cairo, Egypt; 2 Analytical Chemistry Department, Faculty of Pharmacy, Cairo University, Giza, Egypt

**Keywords:** White analytical chemistry, Analytical GREEnness Metric Approach and Software, Veterinary, Sustainable, Tylosin, Sulfadmidine, Trimethoprim, Ternary severely overlapped

## Abstract

Mathematical manipulation technique has proven to be a very powerful tool for efficient processing and handling of highly overlapped spectra. This work introduced two green and sustainable approaches for the successful recovery of the ternary mixture, Tylosin tartarate (TYL), Sulfadimdine (SLD), and Trimethoprim (TRI). The approaches were constructed using three different methods, derivative ratio spectrum zero-crossing method (DRSZ), double divisor ratio spectra derivative method (DDRD), and factorized derivative ratio method coupled with spectrum subtraction (FDRM-SS). The two approaches succeeded in recovering the three drugs (Linearity range achieved were 0.5–5 µg/mL for TYL, 0.3–1.3 µg/mL for SLD, and 0.3–5 µg/mL for TRI), giving convenient standard deviations and satisfactory recovery percentages. The recommended methods have been verified in accordance with (ICH) guidelines. When the results were statistically compared to the official methods, no significant difference was found. Both AGREE—Analytical GREEnness Metric Approach and Software, and White Analytical Chemistry (WAC) RGB model gave scores of 0.93 and 97.2%, respectively, which proved that the approaches were eco-friendly and abiding by the sustainability principles.

**Supplementary Information:**

The online version contains supplementary material available at 10.1186/s13065-024-01220-4.

## Introduction

In recent decades, animal health has received considerable attention due to its crucial importance and contribution to the human dietary system and well-being. Thus, to increase animals’ lifetime and wellness, light was spotted on veterinary antibiotics, and the safety and efficacy of them. Consequently, various antibiotic formulations have been recently fed to the market all of which are of great impact to animal’s welfare. Therefore, the quality control of veterinary formulations has been subjected to numerous controls and regulations, and any study on this very important segment of veterinary drugs is considered significant and lifesaving.

**Fig. 1:**
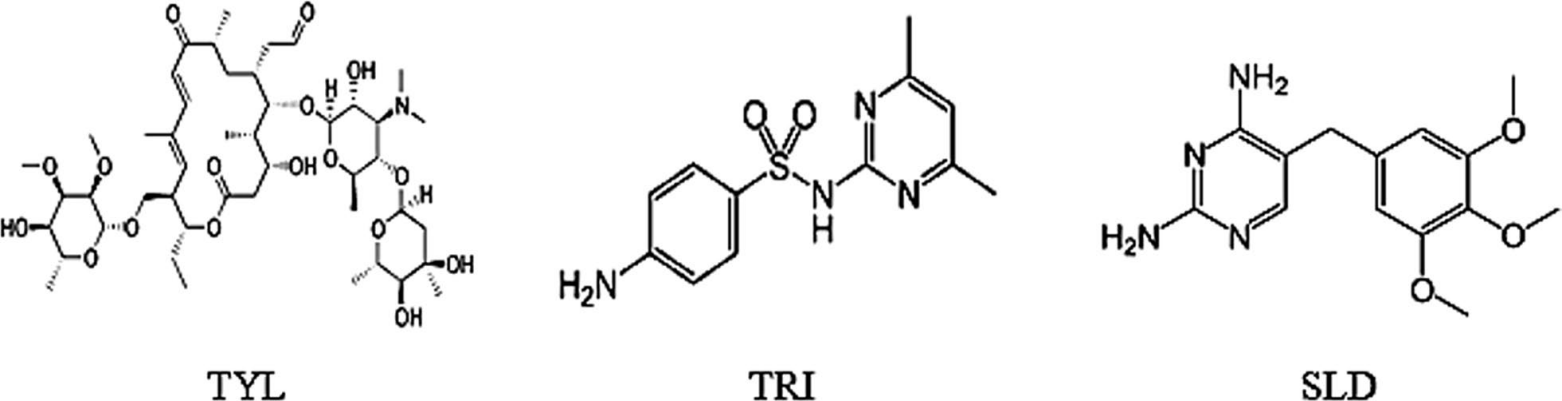
Chemical structures of the three cited drugs

Tylosin tartrate(TYL), having IUPAC name of (4R,5S,6S,7R,9R,11E,13E,15R,16R)-15-[[(6-deoxy-2,3-di-*O*-methyl-b-d-allopyranosyl)oxy]methyl]-6-[[3,6-dideoxy-4-*O*-(2,6-dideoxy-3-C-methyl-a-l-ribo-hexopyranosyl)-3-(dimethylamino)-b-d-glucopyranosyl]oxy]-16-ethyl-4-hydroxy-5,9,13-trimethyl-7-(2-oxoethyl)oxacyclohexadeca-11,13-diene-2,10-dione, as shown in Fig. 1. It is a macrolide antibiotic, Considered a very important antibacterial agent in veterinary formulations [1]. TYL possesses bacteriostatic action against bacteria and Gram-positive mycoplasma [2]. Additionally, it is added to poultry feed as an anticoccidial ingredient. Different methods were described in literature for TYL determination depending on UV–VIS spectrophotometry [3–5], chromatographic methods [6, 7], and electrochemical methods [8, 9].

Sulfadimidine (SLD), having IUPAC name of 4-amino-*N*-(4,6-dimethylpyrimidin-2-yl) benzene sulfonamide, is shown in Fig. 1. It is a sulfa-based drug, used in hindrance and management of various bacterial invasions as actinomyces, and urinary tract infections [10]. Sulfonamides are commonly used in veterinary practice. Several methods of determination were mentioned in literature, including Spectrophotometric methods [11, 12], chromatographic methods [13–15], and electrochemical methods [16].

Trimethoprim (TRI), having IUPAC name of 5-(3,4,5-Trimethoxybenzyl) pyrimidine-2,4-diamine, is shown in Fig. 1. It is a well-known potent metabolic inhibitor of dihydrofolic acid reductase [17]. This drug shows powerful antibacterial activity against strains which are resistant to other frequently used antibiotics, e.g., β-lactams [18].

The three drugs, TYL, SLD, and TRI are found as a ternary mixture in the Egyptian market under the name Tylosin Plus^®^, and veterinarians rely on this formulation regularly for the treatment of various bacterial invasions in large animals. The three drugs are also mentioned in the British Pharmacopoeia [19–21].

As far as we know, there was a chromatographic method in literature describing the determination of the ternary mixture [22–24]. Unfortunately, HPLC was proved to be time-consuming and of high cost, with a lot of chemicals used and waste produced. There is no other work in literature describing an easier and simple, yet green method, like spectrophotometry, for the determination of ternary mixture.

Unfortunately, multicomponent mixtures, including this studied mixture, typically have highly overlapping spectra, which urged the buildup of various methods for determining each studied drug's concentration [25]. Recent incorporated spectrophotometer software that supports mathematical techniques is believed to be an optimal key to resolve complicated spectra, and to get as much analytical data as possible from the unresolved bands [26].

Thus, this study investigated the applicability of using green spectrophotometric methods as; Derivative ratio spectrum zero-crossing method (DRSZ), Double divisor ratio spectra derivative method (DDRD), and Factorized derivative ratio method coupled with spectrum subtraction (FDRM-SS); which are able to resolve severely overlapped ternary mixtures.

Additionally, we aimed to magnify green and white chemistry recognition; we took into consideration sustainability precautions and guidelines throughout the study. Sustainability is a major focus of green chemistry, which seeks to improve resource efficiency and lessen its negative effects on the environment. Specifically, the reduction or elimination of hazardous chemicals is given priority in green analytical procedures. In addition to improving worker safety in the laboratory and aim to reduce waste and pollutant production. Adopting green analytical techniques encourages innovation in technique development, complies with regulatory requirements, and responds to public concerns about environmental responsibility. Green analytical methods incorporate energy efficiency and renewable resource principles, which lead to more conscientious laboratory practices. In the end, this strategy contributes positively to the larger objective of sustainable scientific research [27–29]. The methods’ greenness was evaluated with AGREE greenness tool, where each sector of the clockwise figure illustrating the 12 principles is colored from red to yellow to green, signifying how green each principle is in the methodology. The score is usually closer to one the greener the diagram. The whiteness was assessed by RGB 12 model, where along with the environmental factors, WAC considers two more critical standards that impact the method's quality: analytical (red) and practical (blue). An analysis of whiteness using the RGB color model, which states that the combination of red, green, and blue light beams creates the illusion of whiteness, demonstrates the coherence and synergy of the analytical, ecological, and practical features.

## Materials and methods

### Theoretical background

#### Derivative ratio spectrum zero-crossing method (DRSZ)

In this method [26], a combination containing X, Y and Z that show a severely overlapped spectra, is divided by one of the drugs’ standard solutions, and the DD1 of the resulted spectrum is found (the divisor component is removed by derivatization). To find the concentration of one component at the other component's zero-crossing point, modification is done in the matching regression equation by changing the peak amplitude.

#### Double divisor ratio spectra derivative method (DDRD)

The three-component mixture (X + Y + Z) is divided by (X + Y); an equimolar mixture, then followed by derivatization [26]. In this case, Cz concentration is directly proportional to the derivative at the coincidence points, which represent the minimum and maximum wavelength for pure Z, and Z in the ternary mixture (X + Y + Z). Similar methods could be utilized in estimation of C_x_ and C_Y_ in the ternary combination.

#### Factorized derivative ratio method coupled with spectrum subtraction (FDRM-SS)

This method [30] is a methodology for factorized spectra [31–33] to resolve severely overlapped ternary mixtures spectra (X + Y + Z). In this method, divisor (Z’)’s contribution is negated by derivatization utilizing an appropriate sequence, so Z/Z’ could be negated.

To apply this, an appropriate wavelength is chosen, in which X/Z’ spectrum happens to own a contribution at the zero crossing point of the third drug Y/Z’ (could be zero-crossing or zero-contribution point) to obtain X factorized derivative ratio, in which Px_(λzero point)_=1, DD^1^ of X is divided by its recorded peak amplitude value in this specific wavelength_(λzero point)_.

$$DD^{1} \;factorized\;spectrum\;(FS^{\prime}) = P_{X} /P_{X} V_{(\lambda \;zero\;point)}.$$

The component Z’ is used as a divisor to analyze laboratory-prepared mixtures, where the recorded amplitude is multiplied at a certain wavelength _(λ zero point)_ by the formerly attained X DD^1^ factorized spectrum, derivatization under specified experimental parameter, the DD^1^ spectrum of the elements X(X/Z’) could be found [29].

### Instrumentation

Spectrophotometric measurements were done with the help of an ACER compatible computer that was associated with double beam UV-VIS spectrophotometer model J-760 (Jasco, Japan), using 1.00 cm quartz cells. The Scans were completed between the range of 200–400nm at 0.1 nm intervals, using SpectraManager^®^ software. Sonication was done by an ultra-sonicator (Crest Ultrasonics Corp., NJ, USA).

#### Chemicals and reagents

- Pure samples: SLD, TYL, and TRI with purity 100.06% ± 0.637, 100.45% ± 0.50 and 100.09% ± 0.583, respectively bestowing to the BP official methods, were generously provided by Pharma Swede^®^ Veterinary Company, Cairo Egypt.
- Veterinary formulation: Tylosin Plus^®^ was purchased from a veterinary drugstore in Egypt.
- Reagents: From (Sigma Aldrich^®^, Germany), analytical grade methanol was purchased.

#### Standard solutions

For the preparation of the standard solutions, 10 mg of each component were sonicated in 50 mL methanol for 2 min separately, then each standard solution was filled till 100 mL with methanol to prepare 100 µg/mL, and TYL flask was enfolded with aluminium foil to be sheltered from light.

Each drug's working solution was prepared by adding methanol till mark to a 10 mL volumetric flask that had been filled with various concentrations of the standard solution.

### Procedure

#### Spectral data for TYL, SLD, and TRI

At 200–400 nm, the D^0^ absorption spectra of the three components were scanned against blank (methanol).

##### Factorized spectrum

Using 1 µg/mL SLD as a divisor, the TRI DD^1^ spectrum was divided by the amplitudes obtained at 256 nm. Similarly, SLD DD^1^ spectrum using 1 µg/mL TRI was divided by the recorded amplitudes at 225.1 nm. The computer retained both output spectra.

##### Linearity and calibration graph construction

Aliquots from the 100 µg/mL standard solution equal to 0.3–1.3 µg/mL SLD, 0.5–5 µg/mL TYL, and 0.3–5 µg/mL TRI were taken to 10-mL volumetric flask. Each prepared standard solution was scanned against blank (methanol) at a range of 200-400nm, which were retained on the computer.

*Calibration graph of TYL*

*Approach 1: DD*^*1*^. D^0^ absorption spectra of TYL are divided by 1 µg/mL SLD and plotting against readings at P_max_ 240 nm, then getting the DD^1^ spectrum.

*Approach 2: DD*^*1*^. D^0^ absorption spectra of TYL are divided by 1 µg/mL TRI and plotting against readings at P_max_ 254.7nm then getting the DD^1^ spectrum.

*Calibration graph of SLD*

*Approach 1: DRSZ:* D^0^ absorption spectra of SLD was divided once by 4 µg/mL TYL and its derivative was obtained, and regression equation was constructed by plotting against 272.2 nm.

*Approach 2: FDRM-SS:* Using the divisor 1 µg/mL TRI, the obtained spectrum represented SLD DD^1^. Then to obtain a regression equation, the TRI concentrations were plotted against the peak amplitude at 255.1 nm.

*Calibration graph of TRI*

*Approach 1: DD*^*1*^*.* D^0^ spectra were divided by 1 µg/mL SLD, and first derivative was obtained, then plotted against readings at P_max_ 245 nm.

*Approach 2: DDRD.* The ternary mixture was scanned, along with the solution’ absorption spectra (different TRI concentrations), then by the sum of 1 µg/mL TYL and 1µg/mL SLD absorption spectra (double divisor) to obtain the ratio spectra. Afterwards, their derivatives were obtained, and used for the construction of the regression equation, where the peak amplitude at their coincidence point (236.5nm) was plotted against concentrations of TRI.

### Validation procedures

*Linearity and range*: the proposed methods’ linearity range was valued by checking the concentrations of all three drugs, three times each, and inspecting which range gave best sensitivity.

*Accuracy:* For investigating the proposed method’s accuracy, various concentrations found in the linearity range of each drug were repeated three times implementing the proposed methods.

*Precision:* Validating repeatability and intermediate precision is crucial. This was done by examining 3 separate concentrations of every studied component within its linearity range. This was done by analyzing 0.6, 0.8 and1.0 µg/mL for each drug three times, on a single day, to obtain repeatability. While concentrations 0.5, 0.7 and 0.9 µg/mL for SLD, 1, 3 and 5 µg/mL for TRI and TYL, where investigated on three consecutive days to obtain intermediate precision.

*Specificity*: The proposed methods’ specificity was ensured by inspecting multiple mixtures of (SLD+TRI+ TYL) within the linearity ranges using diverse ratios. Concentrations used for this parameter were mixtures of 2.00, 1.00 and 2.00 µg/mL, 3.00, 1.00 and 3.00 µg/mL, 4.00, 1.00 and 4.00 µg/mL, 5.00, 1.00 and 4.00 µg/mL, and 1.00, 1.25 and 0.50 µg/mL for TYL, SLD, and TRI respectively.

*LOD and LOQ*: The standard deviations of the regression line residuals and the calibration curve’s slope were used to calculate LOD and LOQ, then substituting them respectively in equations:

1 $$\text{LOD }=\frac{(3.3) (\text{SD}) }{\left(\text{slope}\right)}.$$

2 $$\text{LOQ }=\frac{(10) (\text{SD}) }{(\text{slope})}.$$

*Applying the proposed spectrophotometric methods for determining SLD, TYL, and TRI in laboratory-prepared mixtures*

Approach 1: DRSZ method was applied to obtain SLD concentrations, followed by applying FDRM-SS to obtain TRI and TYL concentrations in the ternary mixture as follows:

After obtaining the ternary mixture's DD^1^ ratio spectra and using 4 µg/mL TYL as a divisor, measurements at 272.2 nm were substituted in the matching regression equation to get the SLD concentrations.

The ternary mixture's DD^1^ ratio spectra were obtained, and measurements at 272.2 nm were inserted in the matching regression equation to obtain the SLD concentrations. 4 µg/mL TYL was used as a divisor.

Using 1 µg/mL SLD as a divisor, the DD^1^ of the ternary mixture's ratio spectrum was found. By multiplying the peak amplitude at 256 nm by the previously saved factorized TRI spectrum, the DD^1^ TRI alone was resolved. The peak amplitude at 245 nm from the resolved DD^1^ TRI was substituted in the relevant regression equation to calculate the concentration of TRI. Ultimately, DD^1^ TYL alone is obtained by subtracting the derived DD^1^ TRI spectrum from the ternary mixture’s DD^1^ spectrum. The 240 nm readings in this spectrum were replaced into its matching regression equation to determine the amount of TYL present in the combination.

Approach 2: DDRD was utilized to obtain SLD and TRI, then FDRM-SS was applied to obtain TYL concentrations in the ternary mixture as follows:

An equimolar double divisor (1 µg/mL TYL + 1µg/mL SLD) was used to divide the ternary mixture, and the resulting spectra were then derivatized. The TRI concentration was obtained by substituting readings at 236.5nm in its corresponding regression equations.

To resolve DD^1^ of SLD alone, the first derivative ratio spectrum of the ternary mixture was divided by 1 µg/mL TRI, and its peak amplitude at 225.1 nm was then multiplied by the previously computed SLD factorized spectrum. The peak amplitude at P_min_ (P 255.1) was inserted in the corresponding regression equation to find the concentration of SLD. Lastly, DD^1^ TYL alone is obtained by performing spectrum subtraction, which subtracts the resulting DD^1^ SLD from the ternary mixture’s DD^1^ spectrum. The concentration of TYL in the mixture was determined by inserting readings at 254.7 nm in this spectrum in the associated regression equation.

### Application to the veterinary formulation

One mL of Tylosin Plus^®^ contains 100 mg TYL, 125 mg SLD, and 25 mg TRI, and 25 mg of TRI were added as a standard addition. Firstly, 1 mL of the dosage form was transferred into a 100 mL volumetric flask and completed with methanol till mark. From this, 1 mL is taken in another 100 mL volumetric flask and completed till mark. Finally, 1 mL was taken into a 10 mL volumetric flask and fulfilled with methanol, obtaining a final concentration of 1: 1.25: 0.5 µg/mL of TYL, SLD, and TRI. To obtain the true value of TRI, 0.25 µg/mL of the standard addition was subtracted. Prepared flask was stored away from light.

## Results

Both approaches gave convenient and satisfactory results and recoveries. Regarding Approach 1, TYL accuracy was 100.29, and standard deviation was 0.741. As for SLD, accuracy and standard deviation were 100.77 and 0.179, respectively. Finally, TRI owned accuracy and standard deviation of 100.78 and 0.650.

As for Approach 2, TYL gave results of 100.68 and 1.05 regarding accuracy and standard deviation (standard deviation was higher than that of Approach 1). While SLD gave 100.41 and 0.518 for both results. Finally, TRI’s results were 100.27 and 0.391.

Linearity ranges were 0.5–5 µg/mL, 0.3–1.3 µg/mL, and 0.3–5 µg/ mL for TYL, SLD, and TRI, respectively.

It was clear that Approach 1, where DRSZ, FDRM-SS was utilized, showed lower manipulation steps. Approach 2 had certain limitations as DDRD method requires equimolar divisor concentrations and a coincidence point where measurements are conducted.

### Validation

Method validation was fulfilled with respect to International Conference of Harmonization (ICH) guidelines [30].

#### Linearity and range

Concentrations of the drugs were found to be linear at ranges 0.3–5 µg/mL, 0.3–1.3 µg/mL, 0.5–5 µg/mL regarding TRI, SLD, and TYL, respectively in methanol. The range was satisfactory and allowed manipulation at a wide range, as shown in Table 1.

**Table 1:** Assay parameters and method validation obtained by applying the proposed spectrophotometric methods

Method	TYL		SLD		TRI	
	DD^1^ (TYL/SLD) (240 nm) Approach 1	DD^1^ (TYL/TRI) (254.7 nm) Approach 2	DD^1^ (272.2 nm) Approach 1	DD^1^ (255.1 nm) Approach 2	DD^1^ (245 nm) Approach 1	DDRD (236.5 nm) Approach 2
Range (µg/mL)	0.5–5.0		0.3–1.3		0.3–5.0	
Slope	0.0064	0.0981	0.0536	0.01	0.1566	0.0098
Intercept	0.0005	0.0073	− 0.006	− 0.002	0.0002	0.0013
Correlation coefficient^®^	0.9998	0.9998	0.9999	1.0000	0.9998	0.9998
Accuracy	100.68	100.29	100.77	100.50	100.78	100.27
Repeatability (RSD)^a^	0.089	0.060	0.627	1.425	0.517	1.526
Intermediate Precision (RSD)^b^	0.713	0.562	0.976	0.750	0.880	0.822
Specificity Mean ± SD	100.85 ± 0.800	100.29 ± 0.740	100.77 ± 0.179	100.28 ± 1.3	99.82 ± 1.223	100.49 ± 1.052
LOQ (µg/mL)	0.254	0.500	0.279	0.260	0.057	0.300
LOD(µg/mL)	0.084	0.166	0.093	0.085	0.019	0.100

^a^RSD relative standard deviation of 0.6, 0.8, 1.0 µg/mL for the three drugs were used

^b^RSD (n = 3) relative standard deviation of concentrations 0.5, 0.7, 0.9 µg/mL for SLD, 1, 3, 5 µg/mL for TRI and TYL

#### Accuracy

The accuracy readings were 100.68 and 100.29 regarding TYL, while 100.77, 100.41 and 100.50 for the three methods applied to SLD, and finally 100.27 and 100.78 regarding both methods applied to TRI. As expressed in Table 1, Concentrations were attained with favorable percentage recoveries lying within the accepted range.

#### Precision

Precision plays a pivotal role in ensuring validity and reliability, as it is crucial to ensure that the methods proposed produce consistent results each time they are applied. Speaking of repeatability, results were 0.089 and 0.060 regarding TYL, 0.627, 1.663 and 1.425 regarding SLD, and 1.526 and 1.517 regarding TRI. The results were satisfactory, as clarified in Table 1.

#### Specificity

Another validation factor examined was specificity. Results of TYL were 100.85 and 100.29 obtained from the two methods applied, while 100.77, 99.71, and 100.28 were the specificity results of SLD. Finally, 100.49 and 99.82 were the results of TRI. So, we could say that successful and satisfactory recoveries were obtained, as shown in Table 1.

#### Limit of detection and limit of quantitation (LOD and LOQ)

Usually, acceptable results of LOD and LOQ are associated with 95% probability of obtaining a correct result [34]. Satisfactory results were shown, considering LOQ, 0.500 and 0.254 for TYL, 0.279, 0.050, and 0.260 regarding SLD, and 0.057 and 0.300 regarding TRI.

As for LOD, 0.166 and 0.084 were TYL results, 0.093, 0.016, and 0.085 were SLD’s findings, and finally 0.019 and 0.100 were TRI’ results. Both LOD and LOQ results were shown in Table 1.

### Application to the veterinary formulation

The formulation’s three components, TYL, SLD, and TRI were found in a challenging ratio, 1:1.25:0.25. Therefore, it was not possible to identify the minor component TRI concurrently with the other two components, considering the linearity range of each component. Unless enrichment technique; standard addition [35], was applied. Minor component (TRI) was enriched by standard addition of exactly known concentration of TRI, so that the total response of the veterinary formulation could be successfully replaced in the regression equation to get the accurate concentration of TRI. Subtracting the quantity of standard addition added (amount equivalent to 0.25 µg/mL) from the total drug concentration in the analyzed sample yields the actual TRI concentration present in the dose form.

Table 2 reveals acceptable recovery percentages when applying the proposed methods to the veterinary formulations. Recovery findings like 100.78, 100.74, and 101.90 for TYL, SLD, and TRI, respectively were obtained by the various methods of both approaches. This ensured the success of the proposed methods.

**Table 2:** Analysis of TRI, SLD, and TYL in Tylosin Plus^®^ injection

Tylosin Plus Batch No. 5020118^®^						
Drug	TYL		SLD		TRI	
Method	DD^1^ (TYL/SLD) (240 nm) Approach 1	DD^1^ (TYL/TRI) (254.7 nm) Approach 2	DD^1^ (272.2 nm) Approach 1	DD^1^ (225.1 nm) Approach 2	DD^1^ (245 nm) Approach 1	DDRD (236.5 nm) Approach 2
Claimed Conc	1 µg/mL		1.25 µg/mL		0.25 µg/mL^a^	
Found%^b^	100.78	100.44	100.74	100.40	101.91	102.00
± S.D	± 0.800	± 1.120	± 0.177	± 0.560	± 1.110	± 1.050

^a^After subtraction of 0.25 µg/mL TRI standard addition

^b^All results were the average of three different readings

### Statistical analysis

Statistical analysis was done by comparing the outcomes found by the spectrophotometric methods proposed in this study with the BP official methods [19–21]. After undergoing an ANOVA, t-test, and f-test on students, the computed t and F values were lower than the theoretical ones. This showed that, in terms of accuracy and precision, there was no discernible difference between the outcomes achieved and the official methods. Moreover, ANOVA was also used to compare the recovery claimed from the dosage form using the proposed methods with the method found in literature [19–21]. Both ANOVA results revealed no significant difference, which was illustrated in Tables 3 and 4. Finally, One-way ANOVA testing for the dosage form recovery was compared to the method reported in literature [20–22] (Table 5).

**Table 3:** Statistical comparison between results obtained by the proposed methods and the official methods [20–22]

Drug	TYL			SLD			TRI		
Method	DD^1^ (TYL/SLD) (240 nm) Approach 1	DD^1^ (TYL/TRI) (254.7) Approach 2	Official method^a^	DD^1^ (272.2 nm) Approach 1	DD^1^ (255.1 nm) Approach 2	Official method ^b^	DD^1^ (245 nm) Approach 1	DDRD (236.5 nm) Approach 2	Official method^c^
Mean	100.51	99.82	100.45	99.93	100.23	100.06	100.04	99.88	100.09
S.D.	1.051	0.727	0.500	0.521	0.302	0.637	0.744	0.766	0.583
Variance	1.10400.	0.5285	0.2500	0.2714	0.0912	0.4057	0.5535	0.5867	0.3398
*F* test (5.05)	4.416	2.112	–	1.498	4.448	–	1.628	1.726	–
Student's t-test (1.81)	0.13	1.7	–	0.401	0.587	–	0.110	0.514	–

The figures in parenthesis are the corresponding theoretical values at P = 0.05

The sample number(N) was 5 for all methods

^a^BP Official method includes HPLC method using C18 column and sodium perchlorate (pH 2.5) adjusted with 1 M hydrochloric acid: acetonitrile (60,40, v/v) as mobile phase

^b^BP Official method includes potentiometric nitrite titration

^c^BP Official method includes HPLC method using methanol R and sodium perchlorate in ratios (30:70v/v) adjusted to pH 3.6 using phosphoric acid

**Table 4:** One-way ANOVA testing for the different proposed methods compared to the official methods [20–22] for the determination of TYL, SLD, TRI in pure powdered form

Source of variation	*SS*	*df*	*MS*	*F*	*F crit*
TYL					
Between groups	1.730	2	0.865	1.378	3.682
Within groups	9.413	15	0.627		
Total	11.144	17			
TRI					
Between groups	0.136	2	0.068	0.138	3.682
Within groups	7.400	15	0.493		
Total	7.536	17			
SLD					
Between groups	0.278	2	0.139	0.543	3.682
Within groups	3.842	15	0.256		
Total	4.120	17

**Table 5:** One-way ANOVA testing for the dosage form recovery compared to the method reported in literature [20–22]

Source of variation	*SS*	*df*	*MS*	*F*	*F crit*
TYL					
Between groups	6.919	2	3.459	3.970	5.143
Within groups	5.227	6	0.871		
Total	12.147	8			
SLD					
Between groups	2.299	2	1.149	1.089	5.143
Within groups	6.331	6	1.055		
Total	8.631	8			
TRI					
Between groups	0.703	2	0.351	0.265	5.143
Within groups	7.958	6	1.326		
Total	8.661	8			

### Greenness assessment

**Fig. 2:**
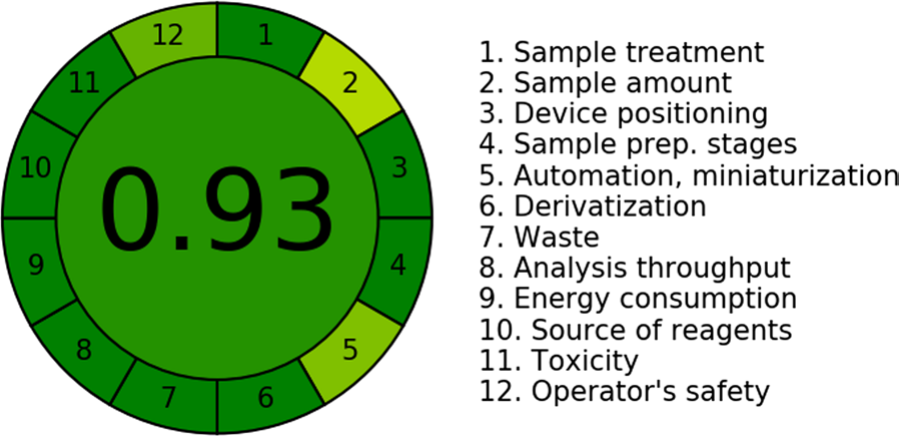
AGREE software results for the proposed mixture

**Fig. 3:**
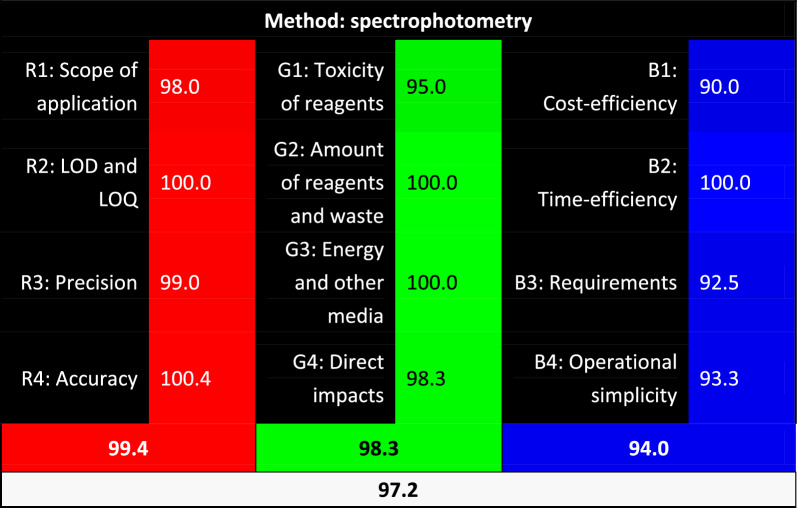
Visualization of the evaluation results according to the RGB method for the proposed approaches

The AGREE score for this study’s proposed methods of GAC was 0.93. The least score was given to point 2 and 5 in the AGREE assessment, referring to sample amount and automation, as shown in Fig. 2. Likely, WAC evaluation model had a score of 97.2, as shown in Fig. 3.

## Discussion

Tylosin Plus^®^ is a reliable antibiotic veterinary formulation used for the treatment of various bacterial invasions in large animals. Its crucial importance and reliance among veterinarians made it vital to further investigate its method of analysis and develop a reliable and easily applicable methodology for its determination.

**Fig. 4:**
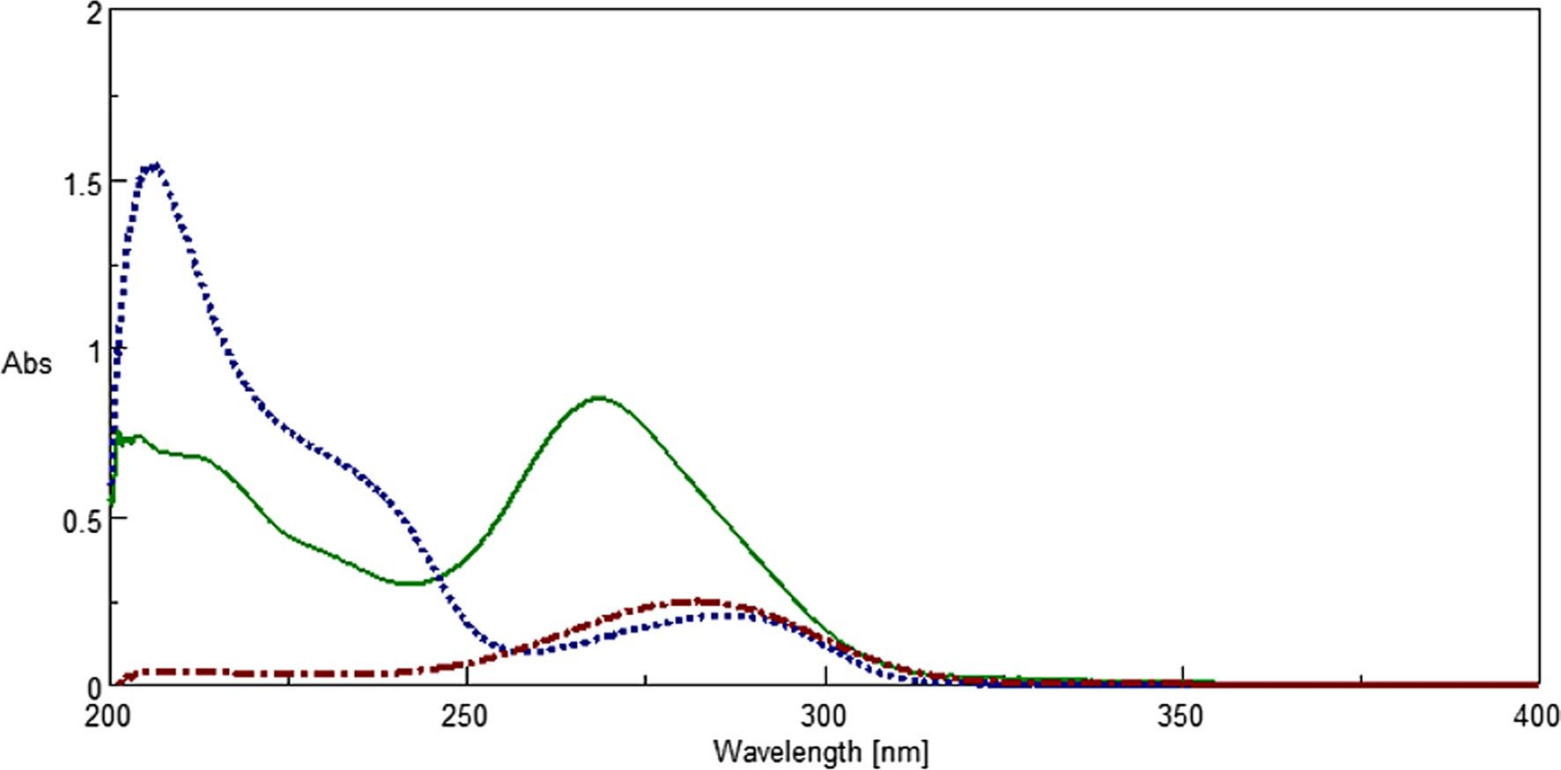
Overlayed Zero order absorption spectra of 1 µg/mL TYL (_._._.), SLD (___), and TRI. **(**. . .) in methanol

The first challenge found in this combination was that the three drugs showed a severely overlapped zero order spectra in the region 200–330nm, as shown in Fig. 4.

There were two approaches demonstrated in this study that successfully recovered all the three drugs in this complex overlapped mixture illustrated in Fig. 1. This was done by utilizing DRSZ, DDRD, and FDRM-SS.

**Fig. 5:**
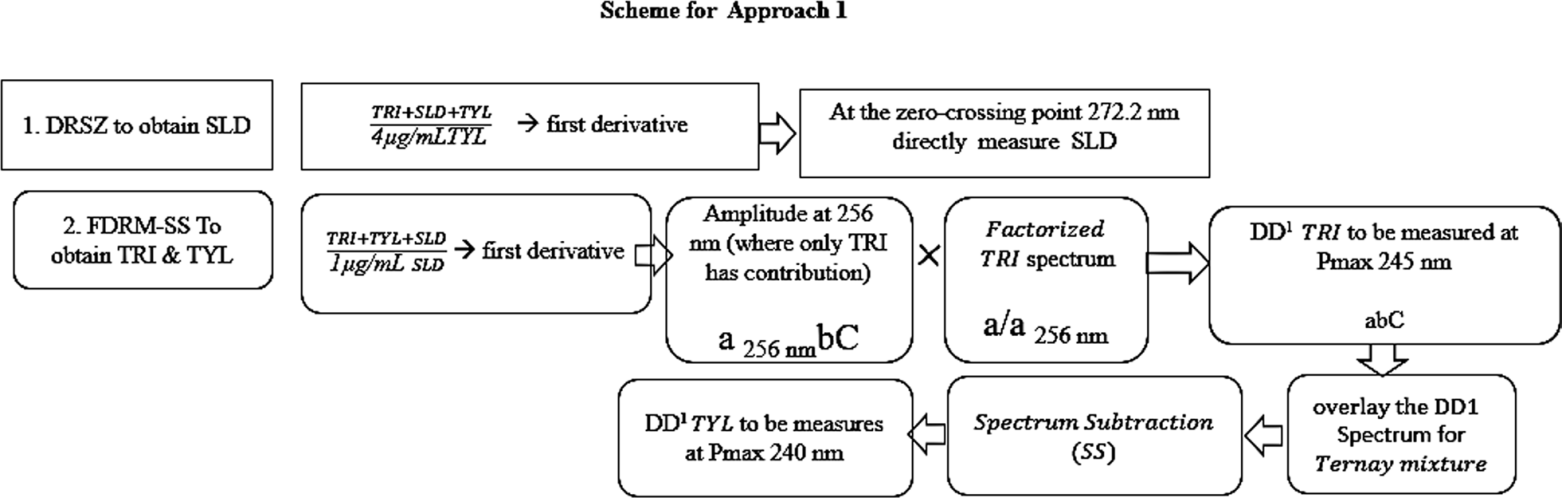
Scheme for approach 1

**Fig. 6:**
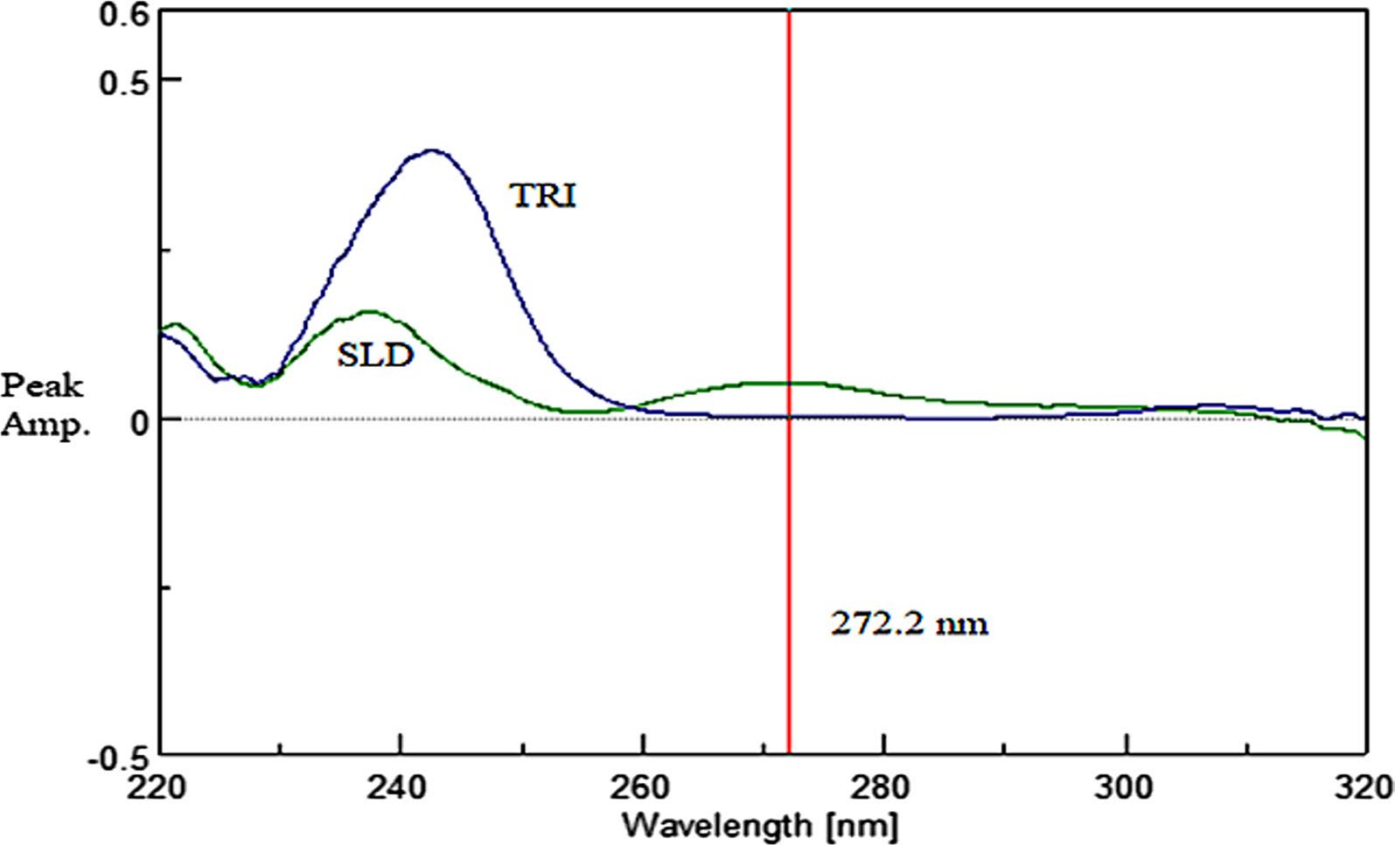
First derivative absorption spectrum of 1 µg/mL of TRI and SLD using 4 µg/mL TYL as a divisor

*Approach 1:* Figure 5 illustrates the whole approach. By getting the ternary mixture’s DD^1^ ratio spectra (SLD + TRI+ TYL) utilizing TYL as a divisor, contribution of TYL in the mixture was cancelled leaving only contribution due to TRI and SLD. SLD was successfully recovered by derivative ratio zero crossing point (DRSZ). As shown in Fig. 6, the SLD and TRI ratio spectra’s first derivative showed a peak at 272.2 nm, where TRI has zero contribution.

Substitution of the peak amplitude at 272.2 nm in the corresponding regression equation, resulted in SLD concentrations alone in the ternary mixture. Several attempts for applying DRSZ to obtain TRI or TYL, but all came to failure, as there was no peak amplitude where the other drug had zero contribution. So, another method was applied to continue resolving TRI and TYL which is FDRM-SS.

**Fig. 7:**
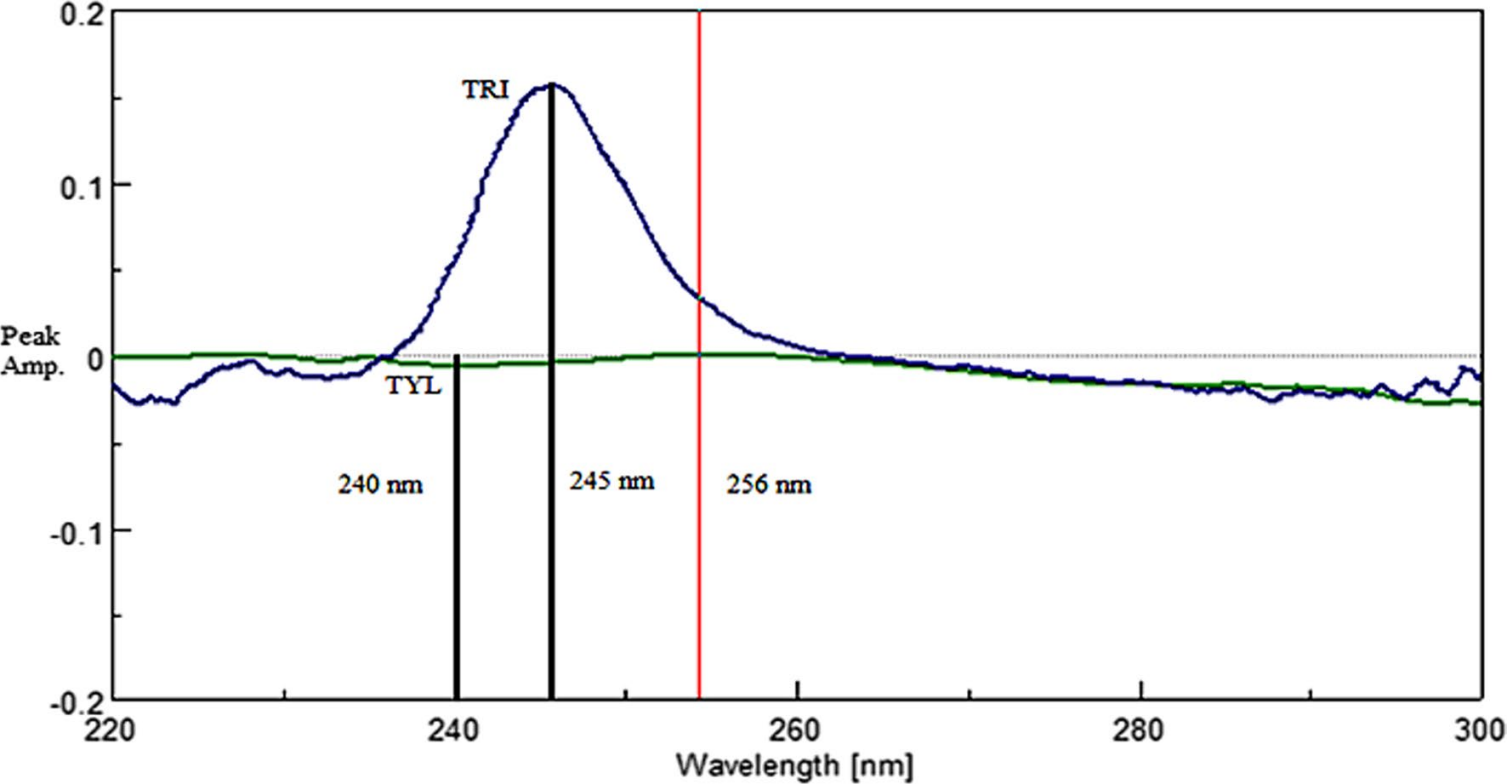
First derivative spectra of both 1 µg/mL TRI and TYL using 1 µg/mL SLD as a divisor

As the concentration of SLD was successfully obtained in the mixture, 1 µg/mL SLD was selected as a divisor for the ternary mixture. Upon derivatization, SLD contribution was cancelled leaving the binary mixture of DD^1^ TRI and DD^1^ TYL. There was no peak amplitude showing zero contribution of the other drug but luckily, there was an amplitude at 256 nm where TRI had a contribution at zero crossing point of TYL, as shown in Fig. 7, therefore FDRM-SS was successfully applied.

Amplitude at 256 nm were multiplied by the factorized spectrum of TRI constructed using this peak amplitude (a/a _256 nm_). The resulted spectrum represented DD^1^ TRI alone, and readings at 245 nm were replaced in its corresponding regression equation. Finally, spectrum subtraction (SS) was applied; after subtracting the resulted DD^1^ TRI spectrum from DD^1^ of the ternary mixture using 1 µg/mL SLD as a divisor, the resulted spectrum should be DD^1^ TYL alone. To get TYL concentration in the mixture, readings at 240 nm, shown in Fig. 7, were replaced in its corresponding regression equation.

**Fig. 8:**
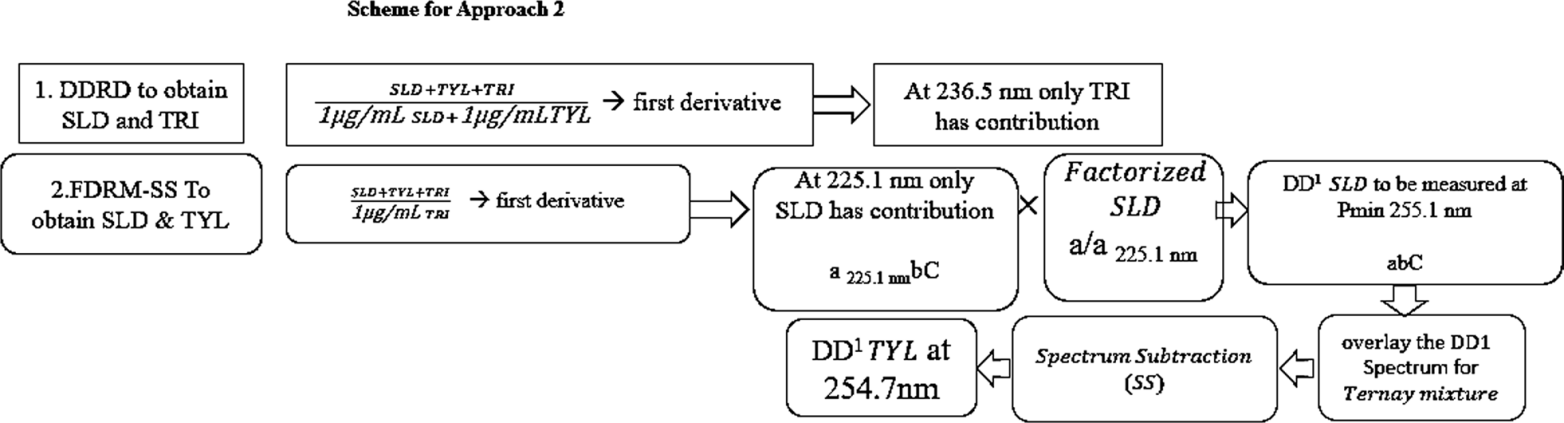
Scheme for approach 2

**Fig. 9:**
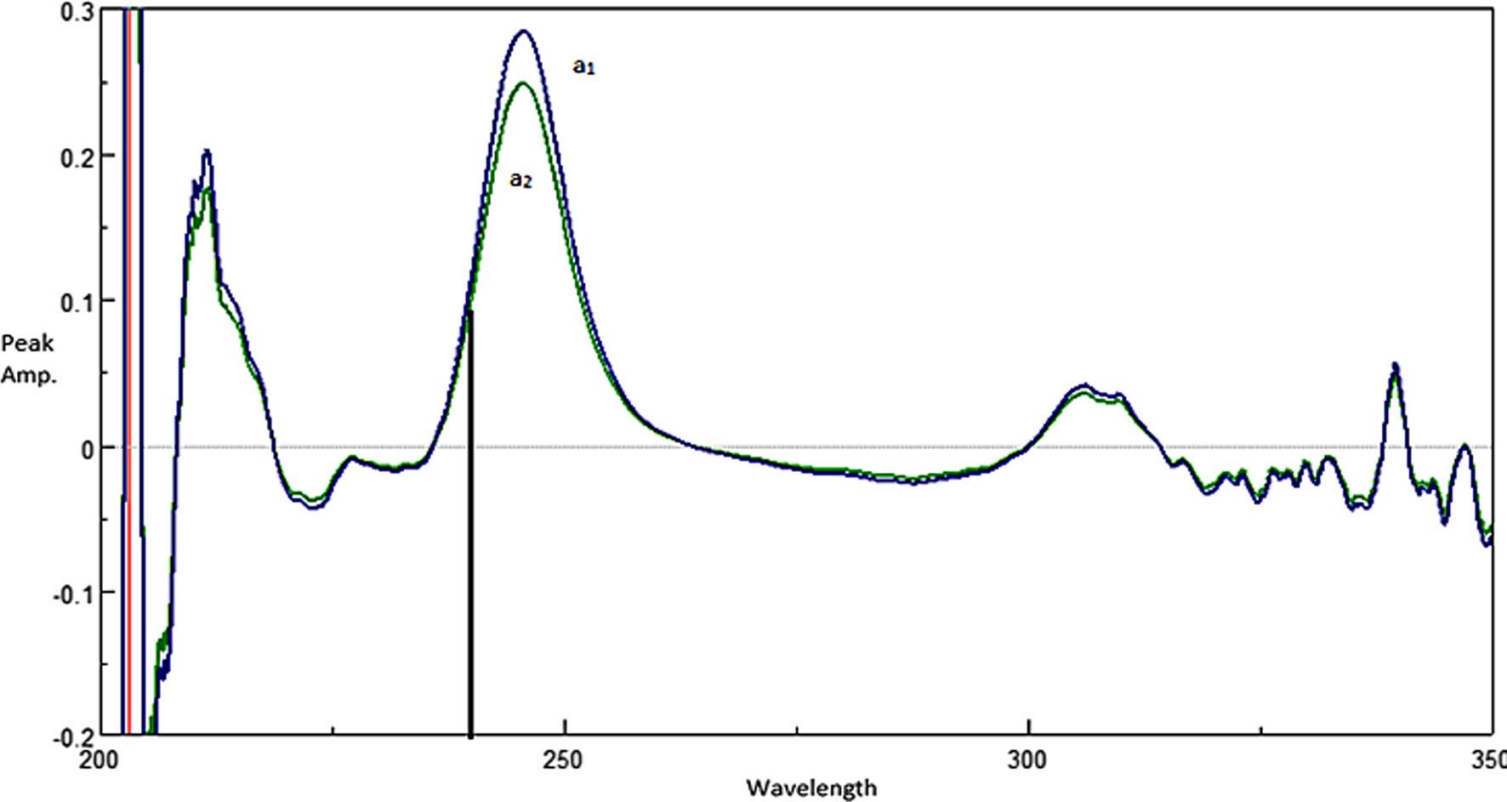
The first derivative of ratio spectra of (a1) ternary mixture of SLD, TRI, and TYL,2 µg/mL each, using double divisor mixture (1 µg/mL TYL + 1 µg/mL SLD), (a2) DD1 of 2 µg/mL pure TRI using the same double divisor, showing coincidence point at 236.5 nm

*Approach 2:* Figure 8 illustrates the whole approach. The DDRD method was investigated as another approach and found to be successfully utilized to obtain TRI. The ternary mixture was divided using an equimolar double divisor mixture (1 µg/mL TYL + 1 µg/mL SLD), and then derivatized. Figure 9 demonstrates this process, where readings at the coincidence point of 236.5 nm were inserted in the relevant regression equation to obtain TRI concentration.

Trials were conducted to obtain TYL or SLD using DDRD but with no success, so FDRM-SS was used to obtain the other two drugs under investigation. FDRRM-SS was furtherly applied by obtaining SLD then subtracting the obtained spectrum from ternary mixture to obtain TYL.

**Fig. 10:**
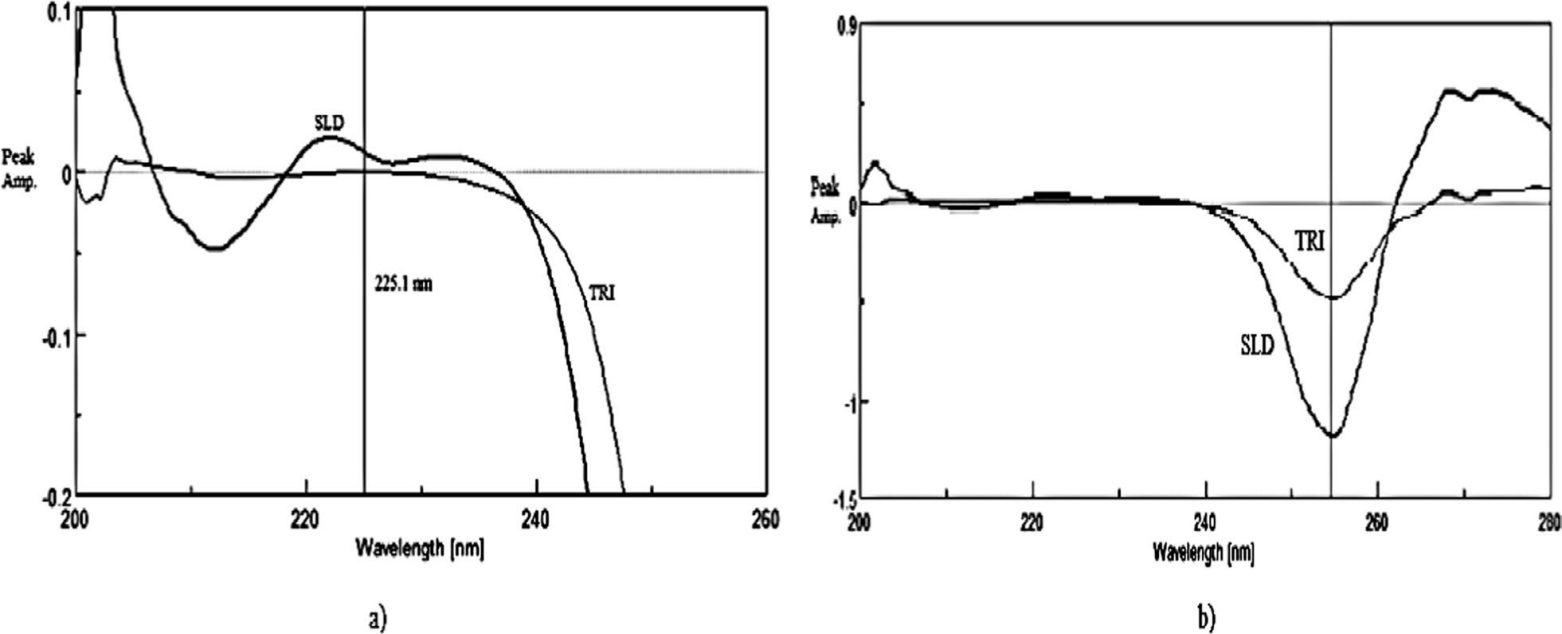
First derivative spectra of both SLD and TYL using 1 µg/mL TRI as a divisor, (**a**) zoom in on the region from 210–240 nm (**b**) zoom out to show the region from 240 to 280 nm showing SLD with P_min_ 255.1 and TYL with P_min_ 254.7

As shown in Fig. 10a, first derivative absorption spectrum of SLD using 1 µg/mL TRI as a divisor owned a shoulder where there was zero contribution of TYL at 225.1 nm. When the ternary mixture (SLD+TYL+TRI) was divided by 1 µg/mL TRI and the first derivative was obtained (TRI was cancelled), readings at 225.1 nm were multiplied with the factorized spectrum of SLD. The resulted spectrum represented DD^1^ SLD alone, and readings at 255.1 nm were replaced in its corresponding regression equation. Finally, SS was applied; after subtracting the resulted DD^1^ SLD spectrum from DD^1^ of the ternary mixture, the resulted spectrum would be DD^1^ TYL alone. To get TYL concentration in the mixture, readings at 254.7 nm were substituted in its corresponding regression equation, as shown in Fig. 10b.

Approach 1, where DRSZ, FDRM-SS was utilized, showed lower manipulation steps. Approach 2 had certain limitations as DDRD method requires equimolar divisor concentrations and a coincidence point where measurements are conducted. As a conclusion, and by taking into consideration the whole process of both approaches, the second approach was recommended as it was more accurate and reliable.

### Greenness assessment

It is obvious that nowadays, within all the uprising environmental drastic changes and warning signs of environmental fatigue, applying green chemistry [36] seems a must. The responsibility and burden of awareness, saving and looking after the environment mainly falls on the shoulders of scientists and researchers. We believe that all researchers should ensure full compliance with the greenness requirements and guidelines in every study made. For instance, it's critical to replace conventional pharmaceutical analysis techniques with eco-friendly approaches with comparable compliance properties, because the former rely on extensive ingestions of many hazardous and organic solvents. This study abided by the greenness guidelines all through the process [37–39]. Moreover, AGREE, one of the most recent greenness assessment instruments, was used to assess the suggested approaches' greenness level [40].

#### Analytical GREEnness metric approach and software (AGREE)

AGREE is a comprehensive and simple assessment method which among all the greenness assessment methods, only AGREE includes all twelve theories of Green Analytical Chemistry [41]. The greenness profile of this study was also assessed using the AGREE calculator, and the predicted AGREE score involving all 12 different concepts of GAC for this study is shown in Fig. 2. The scores more than 0.75 indicate that the method is of greenness for the routine drug determination. Moreover, a 0.50 score implied the method is fit for routine drug determination. Finally, scores under 0.50 indicated the unacceptableness of the proposed method. The AGREE score for this study’s proposed electrodes of GAC was 0.93. These principles encompass reducing or eliminating hazardous compounds, minimizing waste generation, optimizing energy usage, and enhancing safety as well as health. It has a great commitment to environmental responsibility along with sustainability.

The best scores were owned by the sample treatment and energy consumption as they were within greenness limitations, along with all of sample preparation, derivatization, waste, and toxicity as there was none of them. A medium score was given to point 12, referring to toxicity, as methanol is highly flammable. The least score was given to point 2, referring to the sample amount because, according to the second principle of GAC, a minimal sample size and a minimal number of samples are goals. Also point 5 refers to automation, as the method is semi-automized, miniaturized.

The score demonstrates a dedication to sustainability and furthers the better recognized goals of analytical chemistry.

#### WAC evaluation model

White Analytical Chemistry can be conceptualized as a green approaches’ expansion, with an advantage of evaluating whether the proposed method is “fit-for-purpose”, a logical goal all studies should strive for [42]. The approach additionally includes questions about the economical aspect as well as sustainable development (SD), so in other words, it reinforces greenness to go hand in hand with economical value and sustainability.

This new approach is represented by a Red-Blue-Green (RGB) model, in which the 12 new principals are divided among those three colors. “Green” area is related to the greenness, under which four parameters are examined. Then there is the “Red” area, which is related to analytical performances, also grouped under it four parameters. Then finally the area “Blue”, where there are four parameters grouped related to practical benefits.

This study attributed convenient scores for each of the 12 parameters according to the considerations of the authors of WAC. The scores are out of 100, given to a method of zero waste or energy consumption (Green area), a perfect hand-held instrument or easiness of use (Blue area), and convenient analytical performance (Red area). Consecutively, there is an algorithm model summing up all the scores, giving a final average number of (whiteness), quantifying how much the method fits the WAC principles.

The total scores of both AGREE and WAC were very satisfactory, revealing that the proposed methods are of excellent greenness and that we could safely propose our methods to be applied in the future, without causing any harm or damage to the environment.

## Conclusions

Three methods were conducted to resolve severely overlapped ternary mixtures based on derivative ratio spectra. The conventional double divisor derivative ratio (DDRD), derivative ratio zero crossing point (DRSZ), and the recent factorized derivative ratio method (FDRM). Combination between these methods with spectrum subtraction (SS) could overcome the challenging overlapping spectra of the three drugs. This work represents a green, accurate, time-saving, and successful study used to resolve the severe overlap between three components in veterinary formulation, using various manipulating methods, while maintaining high reproducibility and accuracy.

The method has a benefit over the reported method [22] in that, it was timesaving, adhered to green principles, and didn't demand sophisticated experimental setup, intricate processing, or heavy reliance on organic solvents—all of which are commonly accompanying HPLC analytical procedures. Better recoveries of the dosage form were also found utilizing this studied method.

This work was evaluated for the analysis of the above-mentioned components in various veterinary formulations and mixtures and is therefore considered as an alternative tool for routine testing in quality control laboratories.

### Supplementary Information

Supplementary Material 1.

Supplementary Material 2.

## Data Availability

Data is provided within the manuscript or supplementary information files,or upon request, E-mail : hanoood7@hotmail.com.
