# Supplementary material for: Utilizing two different sustainable and green spectrophotometric approaches using derivative ratio spectra for the determination of a ternary severely overlapped mixture: application to veterinary formulation
Source: BMC Chem. 2024 Jun 26;18(1):118. doi: 10.1186/s13065-024-01220-4 (PMC11209959; doi:10.1186/s13065-024-01220-4)
Supplement: Supplementary file 1 — Supplementary Material 1. [file 13065_2024_1220_MOESM1_ESM.pdf]

Analytical Greenness report sheet

17/04/2024 22:40:04

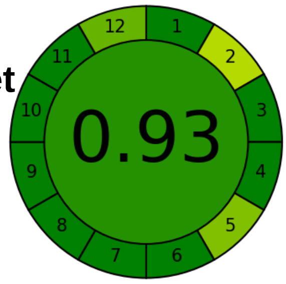

1. Sample treatment
2. Sample amount
3. Device positioning
4. Sample prep. stages
5. Automation, miniaturization
6. Derivatization
7. Waste
8. Analysis throughput
9. Energy consumption
10. Source of reagents
11. Toxicity
12. Operator's safety

Criteria	Score	Weight
1. Direct analytical techniques should be applied to avoid sample treatment.	1.0	2
2. Minimal sample size and minimal number of samples are goals.	0.65	2
3. If possible, measurements should be performed in situ.	1.0	2
4. Integration of analytical processes and operations saves energy and reduces the use of reagents.	1.0	2
5. Automated and miniaturized methods should be selected.	0.75	2
6. Derivatization should be avoided.	1.0	2
7. Generation of a large volume of analytical waste should be avoided, and proper management of analytical waste should be provided.	1.0	2
8. Multi-analyte or multi-parameter methods are preferred versus methods using one analyte at a time.	1.0	2
9. The use of energy should be minimized.	1.0	2
10. Reagents obtained from renewable sources should be preferred.	1.0	2
11. Toxic reagents should be eliminated or replaced.	1.0	2
12. Operator's safety should be increased.	0.8	2
